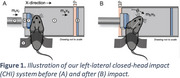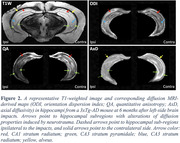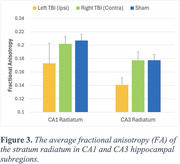# High‐Resolution Diffusion‐MRI Detects Degeneration in Hippocampal Subregions after Neurotrauma in 3xTg‐AD Mice

**DOI:** 10.1002/alz70856_107226

**Published:** 2026-01-12

**Authors:** Ning Hua, Olga Minaeva, Douglas Parsons, Juliet A Moncaster, Elijah Demb, Lee E Goldstein

**Affiliations:** ^1^ Boston University Chobanian & Avedisian School of Medicine, Boston, MA, USA; ^2^ Boston University Alzheimer's Disease Research Center, Boston, MA, USA

## Abstract

**Background:**

Traumatic brain injury (TBI) is a risk factor for the earlier onset of Alzheimer's disease (AD), and the more severe the injury, the greater the risk of developing AD. Given the prevalence of AD in modern society, the possibility that TBI may predispose individuals to develop AD has significant social and economic implications. Therefore, it is important to understand how TBI triggers accelerated AD progression. In this study, we explored how neurotrauma accelerates hippocampal degeneration in a transgenic mouse model of AD using high‐resolution ex vivo diffusion‐MRI.

**Method:**

Unanesthetized 3xTg‐AD mice (*n* = 4) were pretreated with a non‐sedating dose of the analgesic buprenorphine and then subjected to left‐lateral closed‐head impact injury (Figure 1) at 10‐12 weeks of age. At 6‐months post‐TBI, the mice were sacrificed via transcardial perfusion. The harvested brains were submerged in 10% formalin for 24 hours and then stored in Gadavist‐doped PBS (1:400 dilution) until MRI. MRI data were acquired using a 9.4T Bruker scanner and a cryoprobe. Key parameters were TR=300ms, TE=27.7ms, b=3000 (48 directions), and 5000s/mm^2^ (80 directions), FOV=14.30x10.66x7.02mm^3^, Matrix=220x164x108, resolution=65mm^3^. Diffusion MRI was analyzed in DSI Studio and NODDI toolbox. T1‐weighted (T1W) images (resolution=32.5 µm^3^) were also acquired (FLASH) for structural reference. Age‐, gender‐matched 3xTg‐AD mice (*n* = 4) without TBI were used as controls.

**Result:**

Figure 2 shows a representative T1W image and corresponding diffusion‐derived hippocampal maps from a TBI mouse. Compared to the contralateral side, the ipsilateral radiatum of CA1 and CA3 showed decreased quantitative anisotropy (QA) values and increased orientation dispersion index (ODI), the ipsilateral stratum pyramidale showed decreased QA and axial diffusivity (AxD), and the ipsilateral alveus showed decreased AxD. Statistical analysis revealed that the average fractional anisotropy (FA) values in radiatum were lower (CA3, significant; CA1, trend) in the ipsilateral hippocampus compared to the contralateral side of TBI mice or the bilateral sides of control mice (Figure 3).

**Conclusion:**

Our results demonstrate that the hippocampal CA1/CA3 subregions are more vulnerable to neurotrauma. This finding may help clarify the mechanisms underlying trauma‐accelerated AD and suggest that advanced diffusion‐MRI is a potential tool for the early diagnosis of trauma patients at risk of developing AD.